# The Mortal Coil of Covid-19, Fake News, and Negative Epistemic Postdigital Inculcation

**DOI:** 10.1007/s42438-020-00192-7

**Published:** 2020-10-01

**Authors:** Jennifer Rose

**Affiliations:** Queen’s University Belfast, Belfast, Northern Ireland UK

**Keywords:** Fake news, Covid-19, Information disorders, Digital media, Digital technology, Human learning

## Abstract

The Covid-19 pandemic has engendered turmoil around our globe, rendering an urgent need for accurate, truthful information as a life-saving resource for humanity. However, coinciding with this global, deadly pandemic is the proliferation of fake news. While pandemics and fake news are not new phenomena, an unprecedented time in history is presently unfolding when considered with the postdigital era. Digital media enables the prolific repetitious spread of fake news during crises when accurate and truthful information is necessary. Consequently, the ability of humans to discern between fact and fiction diminishes. It has resulted in some people making life-ending decisions based on their exposure to fake news. In this article, I define a primarily ignored and invisible epistemological process at work: *negative epistemic postdigital inculcation*, that, while has been at work with the rise of modern digital media, has primarily become visible because of the interrelationships between implicit learning, Covid-19, fake news, and digital media. While the inculcation outlined in this paper occurs mostly outside of our awareness, I discuss a role for education in helping reduce the ensuing mortal coil of fake news.

## The Mortal Coil^1^ of Covid-19

Pandemics have wreaked havoc on the globe for centuries. The Bubonic plague of the 1300s accumulated a death toll of tens of millions of people. The Spanish flu of 1918 laid millions of people to rest. Similarly, the AIDS pandemic emerging in the 1970s followed suit taking millions of people from the world. Epidemics are an old problem, with new strains and new populations. Coinciding with pandemics are economic, social, and psychological traumas. Many have existed long before this coronavirus outbreak; however, Covid-19 has furthered these devastations worldwide. The World Bank warned that, globally, 60 million people are at risk of extreme poverty due to livelihood and job loss, with businesses permanently closing their doors and non-Western countries bearing economic devastation (BBC News 2020). The consequences of Covid-19 have increased the global battle with mental health, exacerbating existing mental health problems and engendering new cases of psychological distress, including depression, anxiety, and suicide (Carbone and Jorm 2020; Sharma 2020). Mandatory quarantines increased domestic violence because people who were already victims were imprisoned in their homes with their abusers and were isolated from helpful resources and people (Godin 2020). Xenophobia and hate are rampant on a worldwide scale (UNDGC 2020), blighting a centuries-old social problem of inequitably created social identities and exacerbating stigmatization (WHO 2020). Covid-19 has begotten an existence where some homeless people in Calgary, Canada, refuse shelter, food, and a bed opting to hunker beneath bridges or behind restaurants and doorways (Graveland 2020).

Exacerbating the mortal coil of Covid-19 is the viral carnage that has put additional burdens on unsuspecting organizations and institutions. In Ecuador, for example, decomposing Covid cankered corpses lie at home in tropical temperatures wrapped in bedsheets and plastics because the already burdened hospitals are over capacity rendering them unable to accept more dead bodies (Otis 2020). Nauseating stenches fill homes’ air, forcing some family members to place their familial cadavers on sidewalks and streets awaiting pickup by someone from the public health care system (Otis 2020). Some bodies were left in open containers outside packed city hospitals, leaving families to rummage through the corpses searching for relatives (Dupraz-Dobias 2020). Apocalyptic and harrowing scenes proliferate as the city of New York delivers freezer trucks to a funeral home to stack and store the excess decomposing corpses with repulsive stenches emanating from their unrefrigerated body storage (Jackson 2020). Family members are buried without funerals for fear of becoming infected (Amante et al. 2020). Quarantine laws take precedence over bereavement and grief.

Illness has befallen over 25 million people worldwide, with the number increasing daily. High numbers of people infected with the disease do not capture the virus’s insidiousness because people may be carriers without symptoms. At this point in time, over 850,000 people have lost their lives globally, leaving behind rotting bodies and suffering for their survivors. For many of us, our imaginations are surging with fear and anxiety with the stress of becoming ill intensifying distress. The mortal coil of Covid-19 has devastated the continents of our globe, sparing only Antarctica. It may vary between locations; however, it is present and works its destruction primarily hidden from our geographical view. While these examples of the mortal coil of Covid-19 may seem disparate, they raise pressing, paramount, and consequential questions about how we can protect ourselves and others from this deadly virus.

In response to the Covid-19 pandemic, international organizations such as the World Health Organization (WHO) and United Nations (UN), and local governments have put considerable efforts into providing populations with reasoned evidence for the following enjoinments: lockdowns, quarantines, physical distancing, hand sanitizing, or mask-wearing. Additionally, given that Covid-19 is a new virus about which we continue to learn, the WHO has attempted to deliver accurate information at a particular timestamp in history. Possession of accurate and truthful information on protecting oneself and others from a global invisible, rampaging, and deadly virus is undoubtedly one way to encourage humanity’s survival during a pandemic.

However, another old problem, one that is complicit with the world’s historical pandemics and continues to manifest today, is fake news. During the bubonic plague, fake news, for instance, which primarily spread through hearsay, purported that Jewish people were intentionally spreading the plague by ‘poisoning wells, rivers, and springs’ (Clamp 2020). This resulted in many Jewish people being murdered and tortured (Clamp 2020). While pandemics and fake news are not new phenomena, singularly or collectively, what is novel about the current Covid-19 pandemic is that it is the first global pandemic since the rise of modern social and digital media. The global SARS outbreak in the early 2000s occurred before the development of social media sites such as Facebook or Twitter, and digital media only began its transition to digital from primarily paper media later throughout the last two decades (Grabowicz 2014). Covid-19, fake news, and digital and social media, which I will refer to as digital media throughout, have collectively created an unprecedented social time in history. When access to life-saving information is essential, digital media has the potential (and has) enabled the spread of accurate and truthful information online that has helped many people stay safe during this pandemic. However, fake news and its expeditious diffusion through digital media have produced problems for populations. As I will demonstrate shortly, digital media enables the proliferation of fake news (MacKenzie et al. 2020), and does so with an ease that did not exist during previous pandemics.

Covid-19 fake news has diffusedits way through the web of digital media, engendering terms such as ‘infodemic’ by the WHO and ‘disinfodemic’ by UNESCO. UNESCO (2020) even stated that Covid-19 engendered a

parallel pandemic of disinformation that directly impacts lives and livelihoods around the world. Falsehoods and misinformation have proven deadly and sowed confusion about life-saving personal and policy choices.

The question that arises is how does fake news infiltrate our imaginations to the point of infusing it with catastrophic confusion and provoking beliefs of epistemic clarity so that some of us behave in ways that are deadly towards ourselves or others? My claim is that there is a largely ignored, invisible epistemological problem at work that contributes to and comprises the epistemological mayhem of the mortal coil of Covid-19, what I term as: *negative epistemic postdigital inculcation*. Negative epistemic postdigital inculcation is constituted by the interrelationships between our implicit learning, fake news, and digital media. It occurs when we are repetitiously exposed to fake news enabled by digital media. I will expand on this concept shortly; however, first, the concepts of fake news and information disorders require clarification.

## Fake News and Information Disorders

The term ‘fake news’ surged into mainstream media and social networking sites during the US 2016 election campaign. The 45th president declared media that was disagreeable or unsupportive of him, or his political agenda, fake news. The semantics behind fake news is well documented throughout history. False or propagandic new stories have misled or misdirected our minds from either actual or truthful accounts of events and information for centuries (Tattersall and Nevraumont 2018). While the specific terminology of fake news surfaced to discredit truthful news, the neologism has stayed within our imaginations worldwide. Fake news is often used as an all-encompassing term for any information that is false, misleading, or containing partially reported truthfulness. While the term has officially been added to online dictionaries (See online Oxford Learner’s Dictionary,^2^ Cambridge Dictionary,^3^ and Collins Dictionary^4^), the terminology is broad and nonspecific when attempting to identify information disorders (Wardle and Derakhshan 2017). Information disorders are primarily conceptualized by the lack of accuracy and transparency of information and their focus on the messenger’s intention. For instance, misinformation is characterized by a messenger unknowinly spreading and disseminating false or misleading news. Disinformation is defined by a person knowingly and intentionally disseminating inaccurate information. Malinformation manifests when a person uses truthful information with the intention to cause harm (Wardle and Derakhshan 2017). Each of these information disorders illuminates the intention behind the spread of misleading, fabricated, misconstrued, impostered, manipulated, false, and inaccurate information (Wardle and Derakhshan 2017).

However, my central concern is with the *effects* of information disorders. I work on the premise that merely asserting that fake news causes harm is insufficient for understanding its insidiousness. Misinformation, disinformation, and malinformation, while they differ concerning the intention of speaker, writer, and purveyor of fake news, all potentially have the same kind of effect on our imaginations. Deceivers or unknowing spreaders of fake news are still perpetrators of the prolific dissemination of information disorders via the World Wide Web (WWW), television (TV), and other digital media worldwide.

My concern in this article is not, therefore, with the intention of the messenger (or creator) of inaccurate information, even though the messenger and creator are catalysts for the dissemination of information disorders and are undoubtedly problematic. And, I will briefly discuss motivations behind the spread and creation of fake news further on. However, my *central* concern is with the effects of fake news purveyed prolifically through digital media and its implicit relationship with our minds. I, therefore, keep and use the term fake news. Let us now explore some examples of fake news and its inherent connection with human beliefs and behaviour.

## Fake News and Human Beliefs/Behaviour

A man living in Treichville, Cote d’Ivoire, recalled some information he had seen online that stated that rinsing one’s mouth with or drinking a ‘reasonable’ quantity of liquor with high percentage alcohol can exterminate Covid-19 before it can infect the body. Consequently, he acquired a high percentage of alcohol liquor, which he drank because he believed that it would protect him from Covid-19. While ingesting a ‘reasonable’ quantity of alcohol may not be harmful to people, placing aside alcoholic tendencies, it does provide people with a false sense of protection against the virus (Kuwonu 2020). While we do not know the outcome of this man’s fate, his behaviour raises questions about why he believed what he read online.

Glatter (2020) documents the effects of the 45th US president’s comments about exploring the usage of injecting disinfectant into the body as a treatment for Covid-19. These words were not without consequences. The Maryland Emergency Management Agency released an alert after receiving over one hundred calls regarding the ingestion of disinfectants as a potential treatment for Covid-19. Similarly, after the president’s comments, appeals to the New York City Poison Control Center about exposure to household cleaners and disinfectants doubled to thirty telephone calls versus thirteen calls in the previous year (Glatter 2020). Calls to the Illinois state poison control increased, with one person reportedly using a detergent-based sinus solution to exterminate Covid-19. Another person gargled a mixture of detergent and mouth-wash in efforts to eliminate Covid-19. The US Center for Disease control further reported that calls to poison centers increased over the previous 2 years because of people’s exposure to disinfectants. The Washington Poison Center reported a 23% increase in calls about exposure to disinfectants than the number of calls in 2019. Glatter debunks the myths of Covid-19 by reminding people that ingesting bleach is dangerous to human health; however, the damage to human imaginations was already done, as many people had already consumed disinfectants. How do people come to believe that ingesting a seemingly dangerous substance will be beneficial to them?

In Phoenix, Arizona, a man has died. His wife is under critical care after the couple orally ingested chloroquine phosphate, an additive commonly used at aquariums to clean fish tanks, after seeing it on TV and believing that it would protect them from Covid-19. After 30 min of ingestion, both experienced dangerous effects requiring admittance to the nearby hospital. Shortly after admittance, her husband succumbed to the additive, and she was violently ill (Brooks 2020).

In India, Purohit (2020) reports on fake news manifesting as fake advice. Advice surfaced on WhatsApp telling people that they would be cured of Covid-19 by drinking lots of water to keep one’s throat moist, avoiding fried or spicy food, and getting sun exposure infiltrated. WhatsApp is India’s primary social media network and has more than 400 million users (Purohit 2020). The number of fake news messages on WhatsApp in India increased to five-six erroneous coronavirus messages daily versus weekly messages. Citizens searched for answers; however, they were confronted with more viral, fake news, and fake videos about Covid-19. Another video from India alleged that the coronavirus was made in a Chinese government laboratory and was created as a bioweapon (Purohit 2020). The video was viewed over 765,000 times. A man from southern India who thought he was infected with the virus committed suicide after watching numerous fake news videos. It was reported that many people believed these falsities, which resulted in deadly consequences. What was the catalyst for their beliefs?

Cellan-Jones (2020) found that 5G UK groups on Facebook with more than 27,000 members provided fertile territory for spreading misinformation. For example, groups opposed to vaccines were also campaigning against 5G mobile phone networks, provoking scare stories that there is a link between Covid-19 and 5G networks. It was reported that members spread the news that Covid-19 ‘is about as bad as a flu or cold’. Covid-19 is ‘not a serious ‘Virus’ and it ‘is a perfect plan to cover up EMG/5G-related illnesses’. Members of the 5G Facebook groups actively shared videos and websites that supposedly connected Covid-19 to China’s 5G implementation. How did these people come to believe that there is a link between Covid-19 and 5G networks? What might the effects of this fake news be on unsuspecting news consumers?

In Iran, over 600 hundred people died, and 3000 were injured and sent to the hospital after drinking ‘neat’^5^ alcohol upon hearing online that it was a cure for Covid-19 (Pleasance 2020). At first, Iranian officials blamed alcohol poisoning solely on social media for ‘convincing’ people to drink alcohol because some people reported it (Malekian 2020). However, some doctors later revised this report to include a bootlegged alcohol problem in Iran. Still, the fact remains that fake news online has contributed to lost lives. How does this happen?

Covid-19 has indeed intensified and effectuated heeding the need for truthful and accurate information, for without precise information for decision-making, we cannot make informed decisions necessary to save each other’s lives. Knowing what is accurate or true is difficult in online environments and engenders a conundrum for decision-making (Rose 2020). While digital media and online environments enable citizens to acquire information that may not otherwise be available or easily accessible, they also present barriers to knowledge acquisition (Rose 2020). Online content can be designed to represent plausible truths for news consumers (Rose 2020). Because some fake news is designed to represent ‘truth’, if one cannot tell if online content is ‘mis’ or ‘dis’ information, news consumers cannot know or rely on that information to assist them in their decision-making. Alternatively, because fake news is rampant, online news consumers may become the sceptics and become epistemic nihilists, believing little, and drown in ambiguity and distrust. Our ability to acquire knowledge from online environments poses a monumental challenge for news consumers in this fake news, pandemic, digital media era.

I suggest there is more to the Covid-19 and fake news discourse than merely providing accurate and truthful information. This perspective indicates that providing accurate and truthful information will rectify the mortal coil discussed above. Since the outbreak of Covid-19, digital media platforms, globally, have been inundated with information about the virus. Social media posts and messages ranged from accurate information about the pandemic to the blatantly false disinformation and misinformation. Discerning between fact and fiction has become confused—blurred for some people, ending with sometimes deadly consequences. Many causes have contributed to the mortal of Covid-19. Substandard infection control, prevention methods, crowding in highly populated countries, unsanitary health and working conditions, poverty causing the inability to access health care, and the lack of available essential health services are complicit in this pandemic’s carnage (Malta et al. 2020). However, I claim an invisible epistemological problem is at work: *negative epistemic postdigital inculcation*. This process provides insight into why some people believe fake news, and why they make decisions based on false information.

## Negative Epistemic Postdigital Inculcation (NEPI)

Digital technology and media have become requisite to daily life for many people during this pandemic. Computers, e-readers, cellular phones, tablets, and many other digital devices have become necessary, if not before the Covid-19 outbreak, then certainly during this pandemic. Those who opted for analogue options before Covid-19 have undoubtedly been coerced into utilizing computers, software, or any digital device and its corresponding media to keep them connected to family, friends, and colleagues and enable them to function through the quarantines and mandatory lockdowns. And, while digital media enhances our daily activity in many ways enabling us to connect and access information as we have never before, perform work, and engage in leisure or daily occupations—when combined with human minds and fake news, it is also insidious because of what I coin as:

negative epistemic postdigital inculcation – the repetitious exposure to false, misleading, or inaccurate epistemic resources through digital media that become tacit epistemological resources and impact on beliefs and behavior.

Fake news has an epistemically distinct characteristic that is negative—that of possessing undesirable, harmful, false, misleading, or inaccurate information. It is distinctly epistemic because it is directly linked to ‘knowing’. When we encounter fake news, and it enters into our minds—and alters our beliefs—what we know is modified. The postdigital illuminates the intimacy of human-technology relationships. Western Modernity has led us to believe that we are autonomous individuals; however, it obscures the implicit associations that we have with our environment, including technology. Inculcations refer to the human sensitivity to learning from repeated exposure to stimuli. Our minds are not impervious; they are permeable, porous, and sensitive to stimuli in our surroundings. Finally, tacit epistemological resources refer to the epistemic resources that we learn from our surroundings, but about which we are mostly unconscious. Resultantly they do not always qualify as ‘knowledge’. While knowledge can be considered an epistemological resource because we utilize it in our decision-making processes, I reserve the word knowledge for phenomena we can justifiably know and use tacit epistemological resources to account for what we cannot necessarily justifiably know. These concepts collectively comprise *negative epistemic postdigital inculcation* and help explain fake news’s effects on our minds in a postdigital era. While *negative epistemic postdigital inculcation* is a phenomenon that has occurred *before* this present-day pandemic, Covid-19 has illuminated its undoubtable visibility because some people’s beliefs and behaviour are directly affected by exposure to fake news through digital media.

## Our Postdigital Intimacy

Digital technology and the media are no longer considered to be unconnected from our social world; they have become embedded in our societies in complex, taken-for-granted ways (Jandrić et al. 2018). While difficult to define, this postdigital era is ‘messy; unpredictable; digital and analogue; technological and non-technological; and biological and informational’ (Jandrić et al. 2018: 897). The boundaries between us, technology, and digital media are now being ruptured. We are no longer viewed as divorced from technology. The need to highlight the postdigital rupture becomes apparent when we consider our intimate relationship with digital media.

Our digital and epistemic lives are intertwined; they significantly and reciprocally affect what we know and what we *can* know. For example, a key feature of digital media such as algorithms is that they can affect digital literacy and the choices we make about what information to heed when performing Internet searches (Bhatt and MacKenzie 2019). This fosters ignorance (Bhatt and MacKenzie 2019), which affects what we know. Additionally, the constraint around what we can know arises when fact-checking fails us because what we see online appears to correspond and cohere with our reality and beliefs, giving an impression of truth (Rose 2020), thereby affecting our decision-making because our knowing is constrained. A further factor complicating our intimate relationship with digital media is that it affects what we do.

Technology and digital media, in general, enable us to communicate with each other globally. It provides us with the tools (computers, other electronic devices, hardware, software, and the WWW) with which to send, receive, and share messages and information. We know that the WWW connects us across continents. We connect to the Internet and use our email, apps, or social media to communicate with people around the world. However, digital media usage is not benign; digital media directly affects what we do, particularly with respect to fact-checking and spreading information. Twitter, for example, was created and designed specifically as an online social networking platform with which we can share information such as personal opinions or status updates (Oliver 2020). Resultantly, its design lacked features to promote the authentication of information because it was initially created to share personal opinions. As Oliver (2020: 27) explains: ‘there is no provision structured into the logic of Twitter for “fact checking”, verification, review or marshalling evidence’. With a click of a digital button, we can expeditiously retweet fact or fiction, and not consider its truth or falsity because the platform enables us to do so. Twitter is not, however, the only digital media platform complicit in our usage and spread of information. The BBC (2019) reported that WhatsApp’s^6^ sharing function provoked the rapid spread of fake news by allowing a single user to forward the same message up to 20 times. In efforts to combat fake news, WhatsApp limited the forwarding of a single message to five times; however, the perniciousness of sharing fake news had already permeated some people’s imaginations and roused mob lynching in India—where WhatsApp first restricted the number of messages shared (BBC 2018).

Our postdigital intimacy refers to the intimate relationship we have with digital media. This intimacy can have significant effects on our epistemic lives and behaviour. Indeed, the mortal coil of Covid-19 fake news illustrates that digital media is complicit with fake news in affecting our decision-making and what we do. But—the question remains, how does fake news enter into and remain in our imaginations? I seek to elucidate that *negative epistemic postdigital inculcation* exposes the traumatic effects of fake news through a digital virality that infuses some imaginations with befuddled beliefs. We know that digital media enables the prolific spread and *repetition* of fake news for news consumers. A fake news story may originate from a single source; however, when it goes viral online, people may encounter it multiple times (Effron and Raj 2020). Repetitious exposure to fake news through online virality can be a human nemesis (our Achilles heel?) because our postdigital intimacy is enabled by a quality that all humans share, implicit learning.

### Implicit Learning in Our Surroundings

Since the time of Enlightenment, we have striven to be rational and logical in how we make inferences, reach conclusions, perform analysis, and engage in rigorous reflection. Human decision-making referred directly to the ideals of rationality and irrationality. However, over time, Reber (1993: 13) explains, it became apparent that people do not make decisions, resolve problems, or arrive at conclusions using enlightenment idealist rational processes. Instead, when making decisions in their daily lives, people *seemed* to be *arational*. They solved problems and made complex decisions but lacked rationality features, such as explicit logic or reason.

It was not so much that decisions were being made that were irrational, it was rather that decisions were being made on the basis of processes that simply failed to take into consideration rational elements. Moreover, importantly, people often did not seem to know what they knew nor what information it was that they had based their problem solving or decision making on. (Reber 1993: 13)

These processes were deemed irrational not because people are, indeed, irrational but merely because their decisions are based on methods that were not understood and did not traditionally fall into the realm of enlightenment rationality. How people make many decisions is not always based on processes that are explicit or consciously thought. They are made by unconsciously utilizing their tacit knowledge and tacit epistemological resources, which is an accumulation of implicit learning.

However, the concern I have is that our decision-making is not a question of whether or not we are rational or irrational beings. It *is* about *what* epistemological resources we have learned that kindles our behaviour. Rationality and irrationality are irrelevant constructs unless we have the knowledge or epistemological resources to utilize in ‘rational or irrational’ ways. We cannot think about ‘nothing’ rationally or irrationally. Without epistemological resources, our psychological processes are metaphysically simply empty constructs. Knowledge or tacit epistemological resources comprise our psychological constructs and are primarily engendered by implicit learning.

Reber (1993: 5) defines implicit learning as:

the acquisition of knowledge that takes place largely independently of conscious attempts to learn and largely in the absence of explicit knowledge about what was acquired.

Implicit learning does not require us to put effort into learning—we merely learn by exposure to stimuli in our environment. We may or may not be conscious of what we have learned until it manifests in our behaviour or speech. Implicitly learning is the route to acquiring tacit epistemological resources. While outside of our conscious awareness, it plays a role in our decision-making processes (Reber 1993).

Our environments, our surroundings are the stimuli for implicit learning and the accumulation of tacit epistemological resources. This is not the nature versus nurture debate, which asks questions about the impacts of hereditary genetics versus the social environment effects on human development characteristics such as personality. I refer only to the idea that tacit epistemological resources are implicitly learned (Reber 1993). Developmental psychology reminds us that we must be taught as infants, but we also learn from being in our environment. From infanthood to adulthood, we learn explicit epistemological resources, those that we are told or actively seek. And we also learn tacit epistemological resources, which we primarily learn without awareness. When we are in our environments and acquire tacit epistemological resources outside our consciousness, we can say that the environment becomes part of our mind. Consider the process of social normalization within societies. It creates a taken-for-granted reality because we internalize unspoken norms, rules, or practices that become a part of everyday life. These norms keep social order, and these are rarely questioned or explicitly observed unless someone breaches the norms. The excoriator may attempt to doubt normalization or that she behaves in largely unconscious ways. However, even walking develops from implicit learning—a feeling of intuition is a feeling derived from the possession of tacit epistemological resources (Reber 1993). We live and breathe a way of life. We engage in rituals and operate on beliefs in which our imaginations are implicitly or explicitly subsumed. Our reverie is awakened—jarred into consciousness because of the alien, extraterrestrials, who exhibit difference from our internalized way of being, attune us to this difference about which we were previously oblivious. At young ages and throughout our lives we absorb and metabolize stimuli in our environments, which shape our desires, aspirations, and knowledge with any structural or social malaise keeping us on track for cultural re-permutations. We reiterate our civilizations’ knowledge and economic organization by filling neoliberal agendas. We render authority by assigning power to some people and positions over others. We normalize gender, racial superiority, heteronormative sexuality, bodily aesthetics, and general ways of behaving. We become shaped with and without our awareness. The environment in which we wander and reside becomes our reality, our truth, and is our fundamental understanding of the world; it becomes etched into our imaginations in motley creations. Implicit learning and tacit ‘knowing’ are integral to our being. This is not to say that there is a definitive distinction between implicit and explicit learning or tacit and explicit ‘knowing’ (Cleeremans et al. 2019). The relationship is convoluted, interdependent, and challenging to discern. While I discuss implicit learning and tacit epistemological resources, I do so with this recognition of complexity.

## Epistemic Inculcation

Implicit learning occurs when we are in our environment because we are inculcated by repetitious exposure to stimuli that create tacit epistemological resources. We implicitly learn and are shaped by repetitive exposure to the stimuli because we are *sensitive to its frequency of exposure*—in particular, in times of uncertainty (Hasher et al. 1977). When we are in uncertain circumstances or ambiguous situations, the more that we are exposed to particular stimuli, the more likely it becomes a reference for judgements of truth or falsity (Hasher et al. 1977). These become normalized and our truth. In uncertain circumstances, repeated exposure to additional stimuli creates new tacit epistemological resources—an implicit epistemic inculcation. Inculcation does not require excessive exposure to stimuli to become a tacit epistemological resource. Our implicit learning occurs even after moderate exposure to stimuli in our environments. Even minimal exposure creates perceptual fluency and ease of processing, which increases judgements of truth (Reber and Schwarz 1999). Tacit epistemological resources, while work as explicit recognition, also work as implicit ‘intuition’—a ‘feeling’ of familiarity (Sundar et al. 2015: 377).

We can explicitly recall and identify a phenomenon, such as a person who drank ‘neat’ alcohol as a virus prevention measure. He was able to explicitly recall hearing that it would safeguard him. Alternatively, we might have an intuition of familiarity about phenomena merely because we have encountered it before, but we are unconscious of the fact that we ‘know’ it. For example, when someone tells us something ‘new’, we may readily accept what they tell us because ‘it just makes sense’ to us. We accept it not because it is truthful or that we can provide reasons for accepting it. We accept it merely because we have had prior exposure to it. Exposure to events or knowledge seeps into our minds and creates a situation where we might intuitively ‘feel’ that something is real or true because we have encountered it before. Or we might be able to explicitly recall where we have learned it. Once we have implicitly learned, the tacit epistemological resources accumulate and create an epistemic familiarity that serves as an invisible baseline for behaviour and truth judgements. The benchmark of tacit epistemological resources, with or without our awareness, is often used in decision-making (when decisions are not explicitly made). The inculcated tacit epistemological resources are what people tend to use to judge information as true when in uncertain epistemic conditions.

The inculcation of tacit epistemological resources is useful for us because it creates an epistemic familiarity that is helpful and benign in our daily life. However, *negative* epistemic postdigital inculcation creates tacit epistemological resources that are human nemesis. When we encounter repeated exposure to false or misleading information through digital media, this *deceitful information* becomes subjectively familiar to us. Therefore, it is more likely to be assessed as true when we re-encounter it, even if we cannot recall being exposed to it (Hansen et al. 2008; Henkel and Mattson 2011). Negative epistemic postdigital inculcation occurs whether or not the information is true, false, or misleading.

Moreover, information that is implicitly learned *first* is preeminent and assigned more truthfulness than new information presented afterwards (Henkel and Mattson 2011). Implications arise from negative epistemic postdigital inculcation because when fake news proliferates *first*, filling our imaginations with its obfuscations, debunking information at a later date will not necessarily correct the detrimental inculcation. Additionally, because of the baseline of the inculcated tacit epistemological resources that we all possess, warning people about fake news can also be problematic.

Freeze et al. (2020) remind us that not all warnings about fake news are accurate or forthright. While valid warnings focus on reducing the spread and acceptance of misinformation and rectifying its damages, intentionally or unintentionally, invalid or less valid warnings may target factual information. An example might be how the term fake news became a *common* feature of our imaginations worldwide and therefore added to online English dictionaries—merely because a politician issued a warning about true news stories to discredit the information. Readily assigning the term fake news to truthful information is worrisome, because some people believe it merely because they were repetitiously exposed to it. Additionally, information warnings can be less valid because they are imprecise, have broad effects, and may encompass fake news and real news (Freeze et al. 2020). Therefore, charges of fake news should be executed with care as warnings can damage the consumption of accurate information (Freeze et al. 2020).

The epistemological interrelationship between implicit learning, fake news, and digital media requires heeding in a postdigital era. Epistemic postdigital inculcation does not discriminate between fake news and knowledge. The inculcation merely enters into our imaginations with or without our awareness through, even minimally, repetitious exposure to epistemological resources enabled by digital media. Indeed, the inculcation of knowledge is desirable; however, fake news messages that bombard us during our daily use of digital media are not benign or helpful. They are messages that can seep into our minds without our awareness and create new tacit epistemological resources. They become a baseline for truth judgements even though the fake news epistemic resources are false. In a pandemic, when we urgently need accurate and truthful information, erroneous truth judgements can lead people to life or death action based on false beliefs. This is not to presume that all people are affected in the same way by repetitious exposure to fake news. It broadly depends on one’s prior tacit or explicit epistemic resources and the order they were bombarded with fake news. However, the persistent exposure to fake news through its proliferation through digital media creates an epistemic familiarity. This means people *are implicitly learning* about the false, harmful ideas presented in fake news—and even more detrimentally, they are acting upon it. Negative epistemic postdigital inculcation creates a situation where false or misleading information *becomes true* merely by exposure and familiarity through digital media.

During this Covid-19 pandemic, citizens around the world have been exposed to fake news through digital media. As discussed previously, fake news has persistently and prolifically been created, shared, and believed as truth by some people. We can say that the people who ingested disinfectants or chloroquine phosphate believed what they heard through digital media, that they would be cured or protected from Covid-19. Otherwise, placing aside mental health concerns, it is inconsistent to knowingly ingest a harmful substance. While not all instances of fake news result in deadly consequences, drinking a lot of fluids or eating spicy foods is not harmful to people, generally. However, suppose a person comes to believe that she is protected from the virus by eating and drinking certain substances by making judgements of truth. In that case, she places herself at risk for not only contracting the virus but also puts others at risk by potentially being a carrier.

Considering negative epistemic postdigital inculcation concerning Covid-19, people are not necessarily behaving irrationally, suggesting that instilling more rational processes will rectify the problem that fake news poses. They are merely utilizing implicit learning processes inherent in every person. They are building their tacit epistemological repertoire, which operates more fundamentally than constructs of rationality. The human need for *explicit* accurate and truthful epistemological resources has become more visible than ever during Covid-19. However, *tacit* epistemological resources are just as necessary (if not more necessary?) to heed. By merely being human, we implicitly learn much more than we can articulate. Our implicit learning does not discriminate between fact or fiction. And, we learn through repetitious exposure to fake news through digital media. Unfortunately, the ensuing negative epistemic postdigital inculcation has left many of us with devastating effects on our humanity in the present-day pandemic.

Negative epistemic postdigital inculcation is not meant to suggest that it is the sole reason behind each person’s decision. The role of a person’s faith, emotions, source credibility, ideology, for example, may also be implicated in the choices people make. However, arguably, each of these is underpinned by implicit and explicit (or a combination of) epistemological resources. One cannot have ideologies without epistemic resources that comprise an ideology. One cannot make credibility judgements without the possession of epistemic resources about what it means to be credible. One cannot have faith without believing in some epistemic resource. And, while there are many theories of emotions,^7^ the cognitive theory of emotions, for example, suggests that emotions arise from how we think about events or happenings in life. We cannot think about ‘nothing’. Even emotions are derivative from epistemic resources; we feel fear and anxiety about Covid-19 because we think about the deadliness that it poses to our safety. However, this does depend on one’s epistemic orientation towards understanding emotions. The problem that arises is that tacit epistemological resources can influence these other sources. We act on epistemic resources, in which we are unconsciously unaware. While there are lengthy arguments to be made for or against additional reasons which may affect people’s beliefs, behaviour, and decision-making in times of uncertainty, and I do not dismiss these arguments, my central aim for this paper is to discern the idea of *negative epistemic postdigital inculcation* because it is a fundamental, invisible process that spotlights the interrelationship between our implicit learning, fake news, and digital media. Consequentially, it poses challenges for societies and education, and it requires attention.

## Why Does NEPI Matter?

Naysayers may argue that negative epistemic postdigital inculcation is *not* the concern that requires attention, but it is the motivating factors behind the creators and purveyors of fake news that ought to be targeted. I do not disagree that the creation and spread of fake news is highly problematic. However, I will demonstrate that this problem of what motivates fake news is subsumed in systemic epistemological barriers that prevent *absolute* rectification. By systemic epistemological barriers, I refer to the social structures which motivate the creation of fake news. By absolute rectification, I refer to the eradication of fake news. Additionally, because social change occurs in lengthy time frames, without urgently addressing negative epistemic postdigital inculcation, our humanity remains at risk for making life and death decisions on false information.

For example, we know that fake news is not a benign epistemic resource because it is riddled with hoodwinking harmful hoaxes that can wither our wits. It aims to dupe us, and when achieved, our epistemic ability to distinguish fact from fiction flounders. Fake news is a distinct epistemic proposition; the term fake news represents something that is epistemically deceptive. However, it is not an entity; it does not have an independent existence. It is interrelated and embedded into the fabric of our societies. The obviousness of this observation is seen in the fact that fake news necessarily requires creators, messengers, and audiences. Without these, fake news does not exist. A creator can create fake news, but it is inconsequential unless shared because the creator has no one to deceive. We can say, fake news necessarily has an interdependent relationship with us—and we are not free from social contexts. Therefore, fake news is *not* an entity, but it *is* necessarily embedded in our social spheres.

Resultantly, fake news has an interdependent relationship with us and our social structures. Many of us are aware of the motivated lying and dupery in politics, the motivated clickbait creators who succumb to capitalistic power, and the motivated hatred, conjured from historically created colonial identities that motivate the prolific deception about the characteristics of some social groups. These are merely three examples of how fake news is intimately related to our social structures. What other social structures might be implicated as a motivating force for fake news? This question is beyond the scope of the article; however, a question that requires consideration is: what are these social structures? This question may seem fundamental, obvious, and well understood, but I will still briefly explore it.

What are these apparitions that occupy our minds and keep us motivated to behave in particular ways? My claim is that they are *systemic epistemological barriers* that prevent us from the *absolute rectification* of fake news. Capitalism, for instance, is *real* because we feel its effects, give it a name, and we have implicitly or explicitly agreed (or been coerced?) to behave according to its dictates; however, it is invisible a disembodied institution that resides in our minds and affects what we do. It is an epistemological resource that is physically imperceptible. Much like the discussion on implicit learning and the development of tacit epistemological resources, social structures such as capitalism develop within us as an epistemological resource and keep us on track for structural re-permutations.

Additionally, social structures are *systemic* because they permeate many of our minds and constitute our societies. These systemic social structures pose barriers to the absolute rectification of fake news. Suppose deceivers do not disconnect from the influence of social structures that motivate them to deceive, intentionally or unintentionally. In that case, fake news will continue as long as the motivating social structure—the systemic epistemological barrier—remains woefully intact. In such a scenario, fake news remains an insidious problem. As we have seen with this current pandemic, exposure to fake news can be deadly.

Furthermore, social change evolves over a long period of time. For instance, and most distressingly, we are still seeing and experiencing the effects of colonialism present-day, which manifests most blatantly in xenophobia—hate, murder, lynching, or ethnocentrism. I will not propose to answer how long social change takes; however, suspecting minds might infer that it might be several ages (or more?) before a positive systemic epistemological change occurs. Negative epistemic postdigital inculcation provides us with a way of understanding the effects of fake news on our mind and decision-making and requires *immediate* attention. If history repeats itself and our systemic epistemological barriers remain intact, we will continuously be exposed to deceptive fake news and it will not be eradicated. As a result, education can intervene to help reduce the harmful effects of fake news.

### The Catch-22 and Education

The catch-22 of negative epistemic postdigital inculcation is that we need to embrace postdigitality during a time like this Covid-19 pandemic when we must quarantine and self-isolate to reduce the spread of the virus. We necessarily rely on digital media even more than before. However, we also need to remove ourselves from the influence of negative epistemic postdigital inculcation or remove fake news from online environments, as the WHO and many countries such as Iran have attempted. Removing ourselves from digital media or removing fake news from digital media still does not ensure that exposure to fake news will be eliminated, although possibly reduced. For instance, in Los Angeles, CA, fake news paper flyers, bearing a WHO seal, told citizens to avoid Asian-American businesses because of the Covid-19 pandemic (Hay and Caspani 2020).

However, if we are caught in a catch-22, perhaps we can use its features to implement an additional strategy for combatting negative epistemic postdigital inculcation. We can continuously flood digital media *early in times of uncertainty* with a *positive* epistemic postdigital inculcation. Negative epistemic postdigital inculcation can be reduced by repetitiously exposing people to *positive* epistemic resources, rather than negative ones. However, this does not guarantee that people *will be exposed* to the positive epistemic resources because they may be immersed in echo chambers and epistemic bubbles^8^ (Thi Nguyen 2020). While positive epistemic postdigital inculcation may be helpful, it still seems insufficient. Not only might the positive epistemic postdigital inculcation not reach the people that it needs to but also people still lack cognizance; people are at persistent peril because they are unaware that they are implicitly learning tacit epistemic resources that implicitly influence their life or death decisions. These are important strategies but are still inadequate for curbing the problem of fake news.

### A Role for Education

Since the fundamental problem of fake news is epistemic, then it is the epistemological resources that require modification. Long-term, education can investigate ways in which we might re-envision and reconstruct the systemic epistemological barriers that currently motivate deceivers to deceive and believers to believe. We have created these epistemological barriers that have motivated worrisome behaviour; we know this because social structures are not figments of magic. So we can alter them. This is not to suggest that education provides the answer or an epistemological change that will occur with ease. Still, at minimum, education ought to systematically explore and present feasible social structure options that may assist societies in decision-making.

Shorter-term (or not so short term?), education needs to urgently intervene; otherwise, it is plausible that fake news might create the new ‘reality’ through negative epistemic postdigital inculcation; it may create the new ‘systemic epistemological barriers’—which if based on false information will kindle more confusion by pervasively mixing falsities with truth (post-truth?). Specific strategies that education might employ are:

- Encourage populations to stop sharing digital media messages in which they are unaware of its origins. We become perpetrators of the problem of fake news when we share information even if we are unaware of its validity and origins. If we do not know where the information derived, we need to end its digital life.
- Encourage people to be cognitively active when they do not have access to validated information. For example, because inculcations can occur outside our awareness, we may utilize negative epistemic resources when we decision-make; the point at which it manifests in our decision-making is the point at which we become aware that we have been inculcated. With heightened life or death stakes, people need to heed their thought processes and what information manifests in their decision-making. When we catch ourselves using information in our decision making, we need to question whether or not we know if it is true or not, and investigate its factualness before acting on it.
- Remind people and spotlight that the very social structures they live by underpin the use of deceptive fake news. We need to question the motives behind what we see online.
- Remind people that our minds require caretaking. Just as we might limit the consumption of nutrient deficient food, we can make choices about the exposure to ‘nutrient deficient’ information. We can make decisions about what to expose ourselves to or not. This task will also not occur with ease; however, we can limit exposure to false information by choosing our sources of inforrmation carefully, and being cognizant about the factualness of messages that bombard us through digital meia.

The prognosis for immediate systemic epistemological change is grim because a complete epistemological re-envisioning and reconstruction is required. Additionally, fake news and deception have been rampant worldwide for centuries, further demonstrating their resiliency. However, an understanding of negative epistemic postdigital inculcation can help education intervene to reduce the ensuing mortal coil of fake news. 

## Footnotes

1 I borrow the term ‘mortal coil’ from William Shakespeare’s play Hamlet, which refers to daily life’s suffering. Covid-19 mortal coil captures the afflictions with which the malady has infected humans—reminding us that we are epistemically bounded, mortal beings.

2 https://www.oxfordlearnersdictionaries.com/definition/english/fake-news. Accessed 5 September 2020.

3 https://dictionary.cambridge.org/dictionary/english/fake-news. Accessed 5 September 2020.

4 https://www.collinsdictionary.com/dictionary/english/fake-news. Accessed 5 September 2020.

5 Neat alcoholic drinks are drinks that serve a glass of alcohol only, without added mixtures or ice.

6 WhatsApp is a digital messaging app owned by Facebook.

7 See Strongman (2003).

8 An epistemic bubble is a social epistemic group, whether online or not, that may accidentally exclude differing voices from the group. On the other hand, an echo chamber is a social epistemic group, whether online or not, that excludes and discredits differing voices that lead to distrust of all other voices except those of the group (see Thi Nguyen 2020).
